# Supplementary material for: DEAD‐box polypeptide 43 facilitates piRNA amplification by actively liberating RNA from Ago3‐piRISC
Source: EMBO Rep. 2021 Feb 8;22(4):e51313. doi: 10.15252/embr.202051313 (PMC8025031; doi:10.15252/embr.202051313)
Supplement: Supplementary file 1 — Expanded View Figures PDF [file EMBR-22-e51313-s002.pdf]

Expanded View Figures

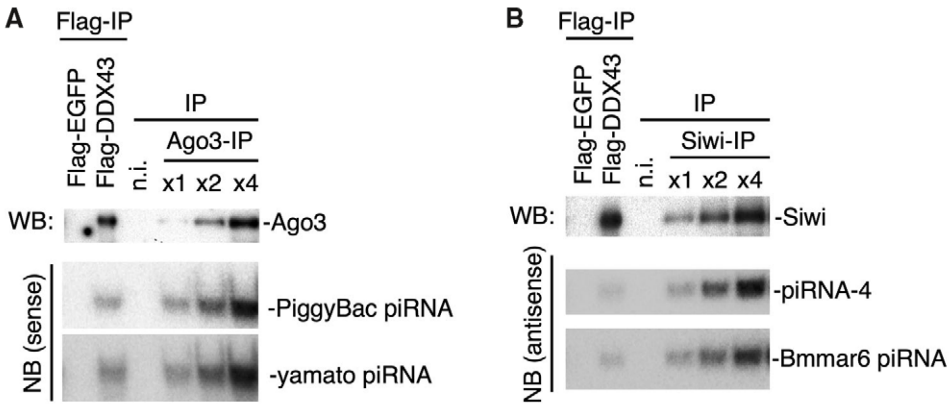

Figure EV1. Analysis of the ratios of piRISC bound to DDX43.

A Ago3 interaction with DDX43 was detected by Western blotting (WB). The Ago3-piRNAs, PiggyBac-piRNA and yamato-piRNA, were detected by Northern blotting (NB).

B Siwi interaction with DDX43 was detected by Western blotting. The Siwi-piRNAs, piRNA-4 and Bmmar6-piRNA, were detected by Northern blotting.

 © 2021 The Authors

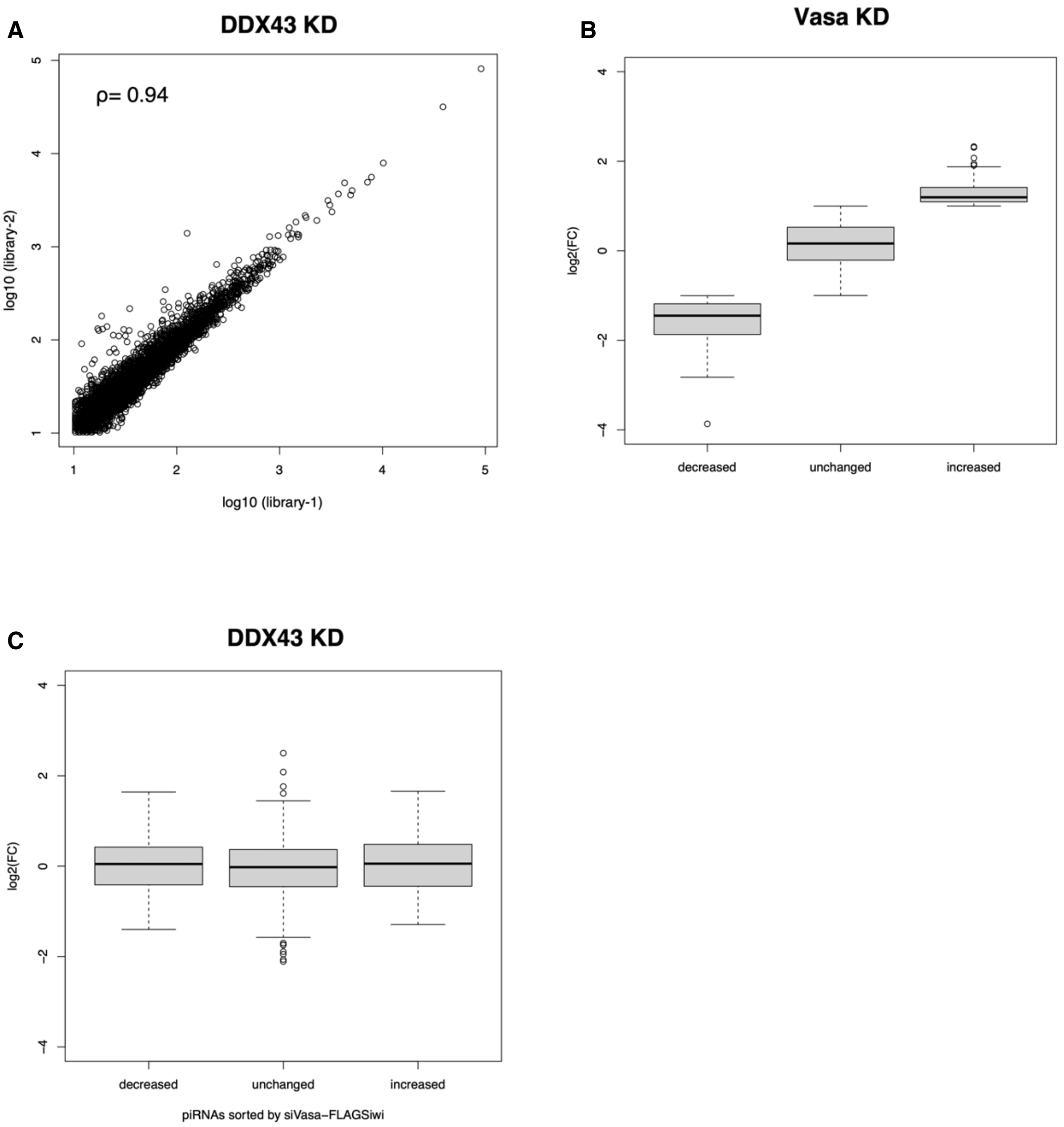

Figure EV2. Comparison of Siwi-piRNA profiles in Vasa- and DDX43-depleted cells.

A Scatter plot of normalized Flag-Siwi-piRNA abundance in two replicate sequencing libraries (library-1 and library-2) generated from DDX43-depleted BmN4 cells (Spearman's Rho = 0.94). Each data point represents a piRNA sequence.

B Boxplot showing the log_2-fold change in the Vasa-KD (RPM)/Control (RPM) ratios. The piRNAs with a reads per million (RPM) ratio ≥ 2, $0.5 < \text{ratio} < 2$, or ≤ 0.5 were categorized as increased, unchanged, and decreased, respectively. Median, first and third quartile, maximum and minimum values, and outliers are presented as the central band, boxes, whiskers, and circles. The combined datasets ($n = 2$, biological replicates) were used for analysis.

C Boxplot showing the log_2-fold change in the DDX43-KD (RPM)/Control (RPM) ratios of piRNAs in the increased, unchanged, and decreased groups as defined in (B). Median, first and third quartile, maximum and minimum values, and outliers are presented as the central band, boxes, whiskers, and circles. The combined datasets ($n = 2$, biological replicates) were used for analysis.

© 2021 The Authors

Figure EV3. *In vivo* reporter assay and *in vitro* unwinding assay.

A Experimental procedure for the *in vivo* reporter assay (see Materials and Methods for further details) used to detect artificial Siwi-piRNA produced by a reporter plasmid encoding the PiggyBac-piRNA target sequence.

B Analysis of the Yamato-based and Pao-based artificial piRNA biogenesis in DDX43-depleted BmN4 cells related to Fig 3C.

C The experimental procedure for the Ago3-cleaved target RNA-unwinding assay (see Materials and Methods for further details).

D *In vitro* target RNA cleavage by Ago3 and fractionation of the cleaved RNAs. n.i.: non-immune mouse IgG. Sup: supernatant. The cleaved target RNA remained bound to Ago3-piRISC upon cleavage.

E Model of step-by-step pre-Siwi-piRISC assembly: Upon cleavage, the 5′-end of 3′-fragment is first bound to Siwi while the 3′-end of the piRNA precursor is still attached to Ago3-piRISC. The 3′-end of the fragment is then released from Ago3-piRISC, producing pre-Siwi-piRISC for Zuc-dependent maturation.

 © 2021 The Authors

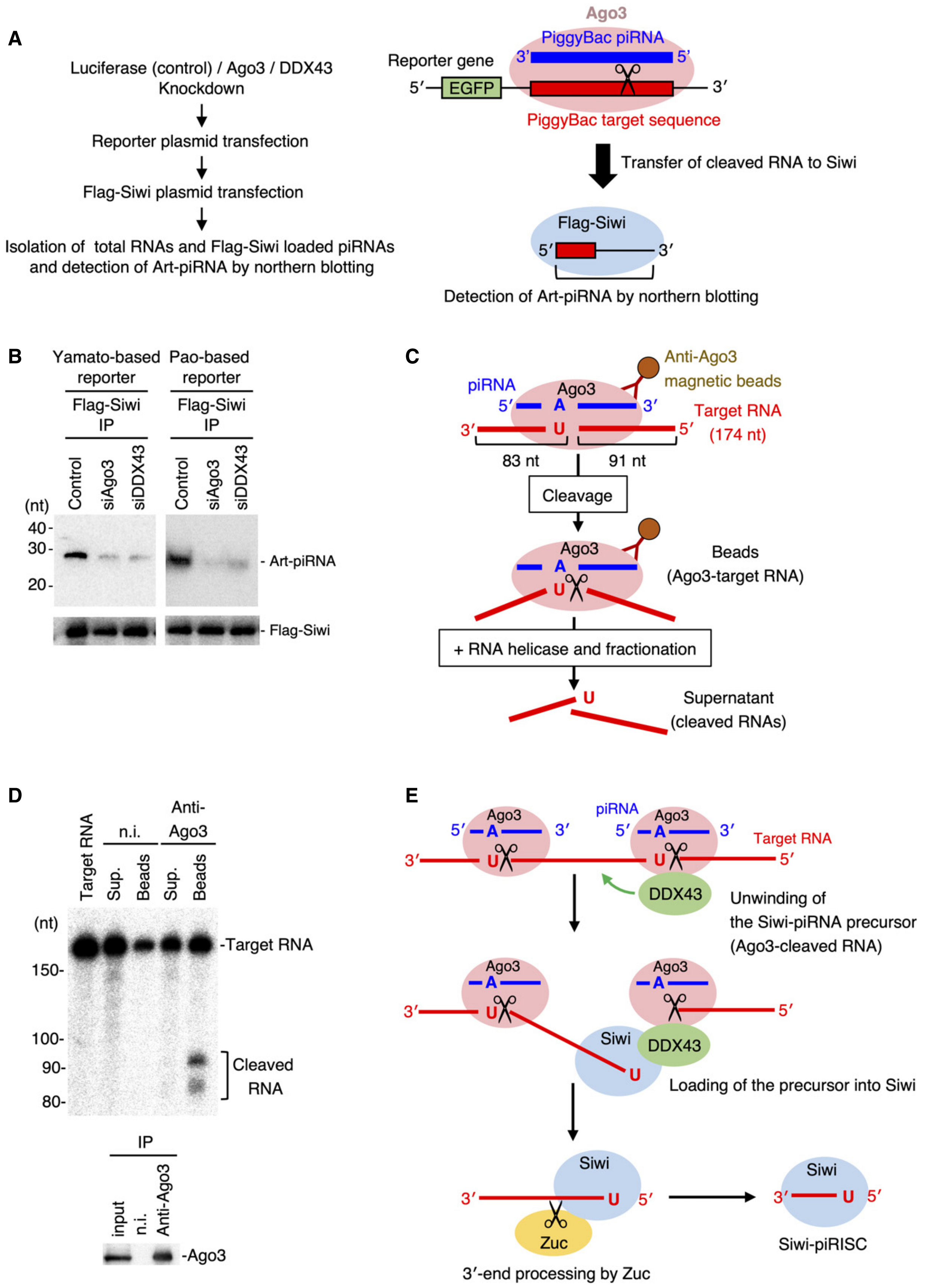
A
Luciferase (control) / Ago3 / DDX43
Knockdown
Reporter plasmid transfection
Flag-Siwi plasmid transfection
Isolation of total RNAs and Flag-Siwi loaded piRNAs
and detection of Art-piRNA by northern blotting
Ago3
PiggyBac piRNA
Reporter gene
EGFP
PiggyBac target sequence
Transfer of cleaved RNA to Siwi
Flag-Siwi
Detection of Art-piRNA by northern blotting
B
Yamato-based reporter
Pao-based reporter
Flag-Siwi IP
Control
siAgo3
siDDX43
(nt)
40
30
20
Art-piRNA
Flag-Siwi
C
Anti-Ago3 magnetic beads
piRNA
Ago3
Target RNA (174 nt)
83 nt
91 nt
Cleavage
Beads (Ago3-target RNA)
+ RNA helicase and fractionation
Supernatant (cleaved RNAs)
D
Target RNA
n.i.
Anti-Ago3
Sup.
Beads
(nt)
150-
100-
90-
80-
Cleaved RNA
IP
input
Ago3
E
DDX43
Unwinding of the Siwi-piRNA precursor (Ago3-cleaved RNA)
Siwi
Loading of the precursor into Siwi
Zuc
3′-end processing by Zuc
Siwi-piRISC

Figure EV3.

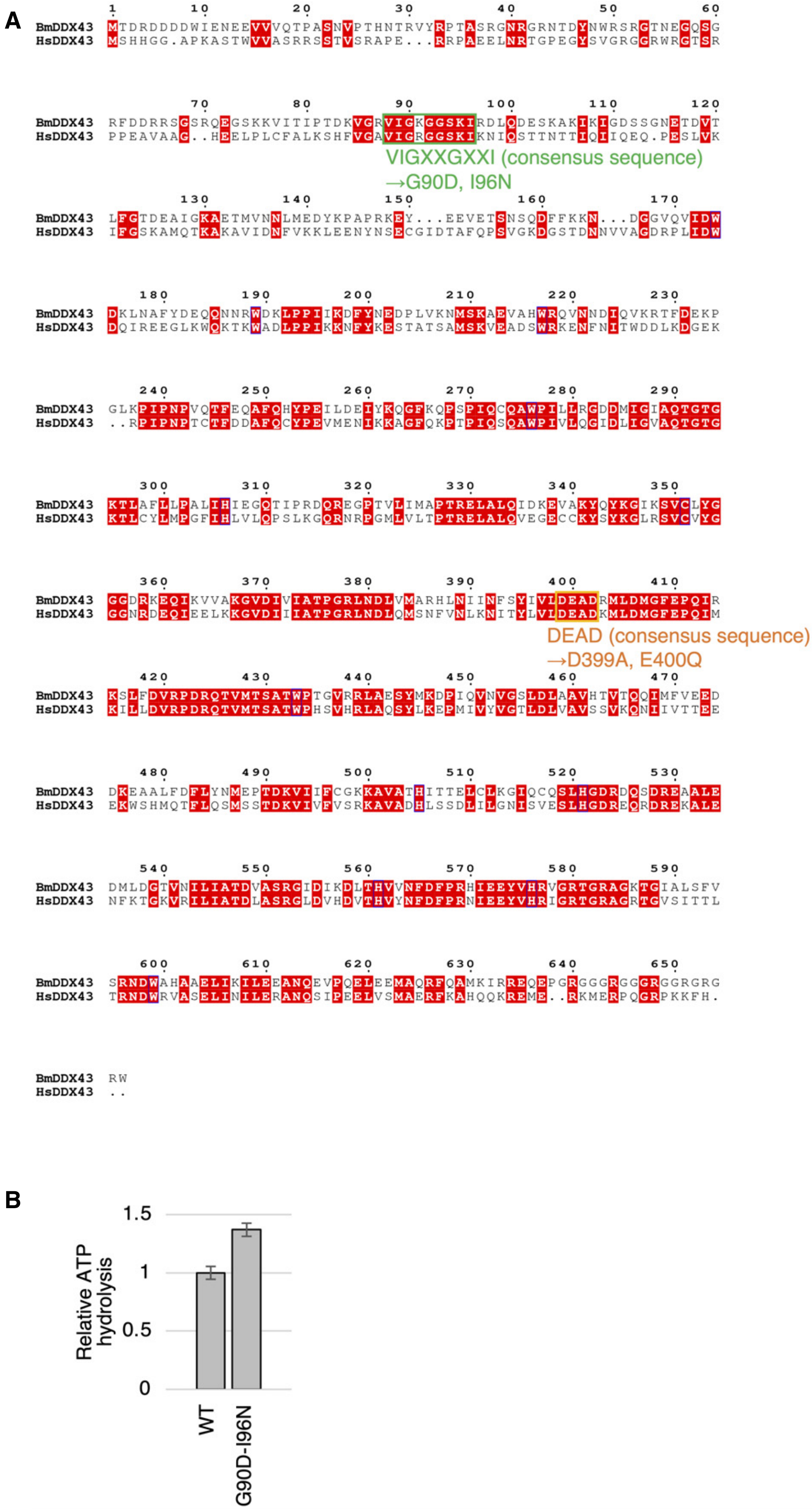

Figure EV4. The consensus sequence in the DEAD and KD domains of DDX43.

A Sequence alignment of human and *Bombyx* DDX43. The conserved motifs of the KH domain and DEAD-box domain are indicated in green and orange, respectively.

B ATPase activity of the DDX43 G90D-I96N mutant (GIDN). Data represent the mean ± standard deviation, n = 3 independent experiments.

 © 2021 The Authors
